# Interpretable Machine Learning of High-Dimensional Aging Health Trajectories

**DOI:** 10.1093/geroni/igab046.2534

**Published:** 2021-12-17

**Authors:** Spencer Farrell, Arnold Mitnitski, Kenneth Rockwood, Andrew Rutenberg

**Affiliations:** 1 Dalhousie University, Halifax, Nova Scotia, Canada; 2 Dalhousie Unversity, Dalhousie University, Nova Scotia, Canada; 3 Dalhousie University, Dalhousie Unversity, Nova Scotia, Canada

## Abstract

We have built a computational model of individual aging trajectories of health and survival, that contains physical, functional, and biological variables, and is conditioned on demographic, lifestyle, and medical background information. We combine techniques of modern machine learning with an interpretable network approach, where health variables are coupled by an explicit interaction network within a stochastic dynamical system. Our model is scalable to large longitudinal data sets, is predictive of individual high-dimensional health trajectories and survival from baseline health states, and infers an interpretable network of directed interactions between the health variables. The network identifies plausible physiological connections between health variables and clusters of strongly connected heath variables. We use English Longitudinal Study of Aging (ELSA) data to train our model and show that it performs better than traditional linear models for health outcomes and survival. Our model can also be used to generate synthetic individuals that age realistically, to impute missing data, and to simulate future aging outcomes given an arbitrary initial health state.

